# Detection of differential bait proteoforms through immunoprecipitation-mass spectrometry data analysis

**DOI:** 10.1038/s41597-024-03394-x

**Published:** 2024-05-29

**Authors:** Savvas Kourtis, Damiano Cianferoni, Luis Serrano, Sara Sdelci

**Affiliations:** Centre for Genomic Regulation (CRG), The Barcelona Institute of Science and Technology, Barcelona, Spain

**Keywords:** Proteome informatics, Proteomics

## Abstract

Proteins are often referred to as the workhorses of cells, and their interactions are necessary to facilitate specific cellular functions. Despite the recognition that protein-protein interactions, and thus protein functions, are determined by proteoform states, such as mutations and post-translational modifications (PTMs), methods for determining the differential abundance of proteoforms across conditions are very limited. Classically, immunoprecipitation coupled with mass spectrometry (IP-MS) has been used to understand how the interactome (preys) of a given protein (bait) changes between conditions to elicit specific cellular functions. Reversing this concept, we present here a new workflow for IP-MS data analysis that focuses on identifying the differential peptidoforms of the bait protein between conditions. This method can provide detailed information about specific bait proteoforms, potentially revealing pathogenic protein states that can be exploited for the development of targeted therapies.

## Introduction

Proteins are widely regarded as the functional units of cells, facilitating functions such as transcription, translation, metabolism and signal transduction. The specific functions that each protein performs are determined by their structure, which is in turn encoded by its amino acid sequence. Mutations to this sequence, as well as post-translational modifications can give rise to proteoforms that expand the repertoire of functions that a protein can perform. Such changes in proteoform function include phosphorylation-based activation, localisation, and mutation-mediated oncogenicity among others^1^.

Recent efforts have attempted a genome-wide cataloging of proteoforms in specific conditions such as blood cell types^2^, which opens the possibility of determining differential proteoform presence. Top-down proteomics has been demonstrated to detect differentially present proteoforms associated with liver transplant rejection^3^. However, despite advances in the field, top-down is still not widely adopted by the community, creating the need for a bottom-up differential proteoform approach.

**Fig. 1:**
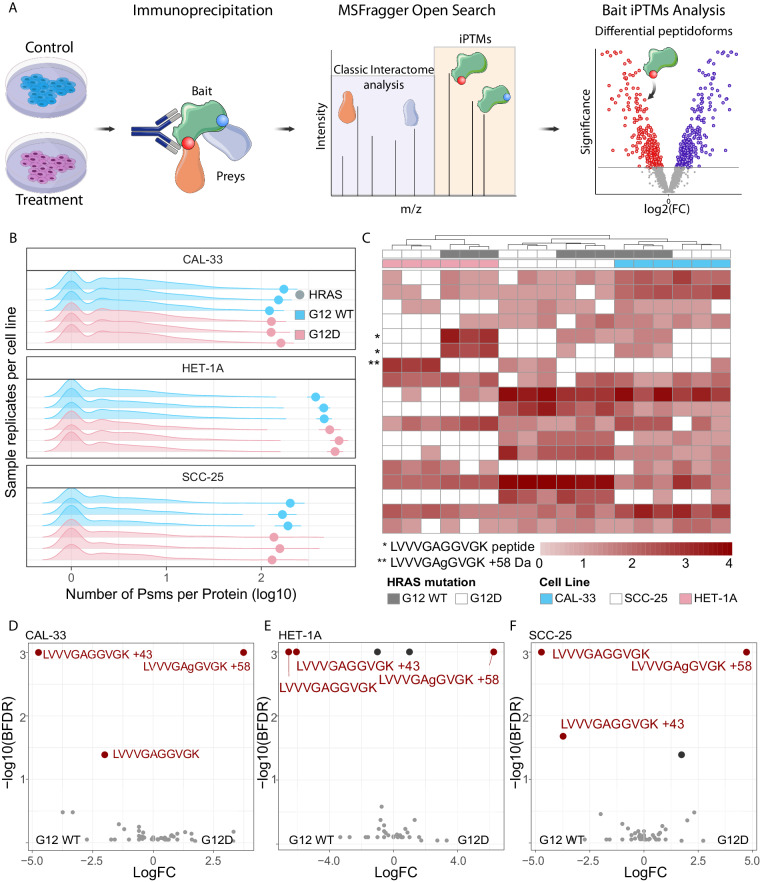
iPTMs detection of genetic mutant peptidoforms. (**A**) iPTMs workflow for analysis of IP-MS data, using MSFragger in Open search with SAINTexpress downstream to identify differentially present peptidoforms between conditions. (**B**) Pre-normalisation PSM counts for prey (density plots) and bait (dots) proteins for paired HRAS WT (blue) and G12D (salmon) samples across cell lines, for each replicate IP-MS experiment. (**C**) Clustering of post-normalised PSM counts for highly abundant peptidoforms belonging to bait HRAS protein. Unsupervised clustering performed with default hclust parameters with peptidoforms not detected in specific samples assigned a zero value following log base 2 transformation. The LVVVGAGGVGK peptide, is highlighted with an asterisk in its unmutated peptidoform, either unmodified (above) or carbamylated (below), while the mutated peptidoform is marked by double asterisk with the lower case amino acid notation highlighting the MSFragger localisation of the +58 delta mass. (**D**) D-F differential bait peptidoforms detected between WT and G12D HRAS in CAL-33 (D), HET-1A (E), SCC-25 (F) cell lines according to the SAINTexpress, defined as BFDR <0.05. Only bait peptidoforms were included in the SAINTexpress analysis.

Here we present a workflow for the detection of differentially present peptidoforms^4^ from classical IP-MS experiments, which traditionally aim to detect the interactome of a bait protein^5^. While classical IP-MS focuses on the differential peptide spectrum matches (PSMs) of ‘prey’ proteins to identify interactors, we propose that the differential PSMs of the ‘bait’ protein could provide differential peptidoform information (Fig. 1A). Our workflow is based on the premise that the bait peptidoforms are enriched by the antibody-based capture of the bait by the protocol, allowing their reproducible detection through MSFragger Open search^6,7^ and differential analysis between conditions using SAINTexpress^8^. Building on previous works that analyse IP-MS data with open search, thus eliminating the need for PTM-specific enriched samples^7,9^, our workflow identifies differentially present peptidoforms of bait proteins between conditions (Fig. 1A).

To test our hypothesis, we decided to focus on cancer-related proteins, reasoning that the detection of disease-specific proteoforms could help in the development of future cancer therapies. We focused on three well-studied proteins: HRas (HRAS) and KRas (KRAS), which are frequently mutated in cancer^10^, and bromodomain-containing protein 4 (BRD4), which promotes cancer-associated transcriptional programs^11^. As these proteins have been extensively studied for their role in cancer progression, their interactomes are available and we were able to investigate whether our analysis pipeline could provide new insights into their functional characterization.

HRAS oncogenic mutations that switch GTPase activity to a constitutively active state alter protein functionality and its interactome^12^. By reanalyzing the pull-down of overexpressed wild-type or oncogenic HRAS, we demonstrated that our workflow can identify the identity and location of the HRAS mutation in an unbiased manner. We therefore sought to identify non-sequence-based proteoforms, such as those resulting from post-translational modifications. To do this, we first focused on the pull-down of KRAS overexpression in the presence and absence of AMG-510, the first-in-class FDA-approved KRAS^G12C^ inhibitor^13,14^. Using our workflow, we identified the residue C118, which is known to facilitate the GTPase activity^15^, as differentially oxidised in the presence of AMG-510.

Finally, we tested whether our workflow can identify differentially present endogenous proteoforms between different cell lines. To this end, we used available data from four different leukemic cell lines and identified differential phosphorylation sites of endogenous BRD4.

The interplay between protein function, its interactome and its proteoform state in a dynamic cellular context is a rapidly growing field of research. In this context, our methodology, which complements interactome analysis with bait proteoform states, can provide novel information about the state of a protein that may be related to changes in its interactome and function, providing a deeper layer of information that can be used to achieve a thorough understanding of protein regulation^16^.

## Results

### Identification of sequence-based proteoforms

**Fig. 2:**
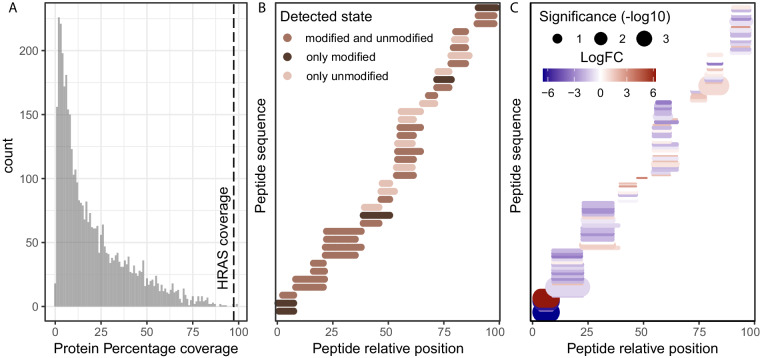
Descriptors of HRAS IP-MS case study. (**A**) Percentage protein coverage for all preys and baits calculated as the percentage of amino acids detected per gene according to the longest isoform, across all HRAS IP-MS samples and all peptidoforms. Dotted line highlight the bait (HRAS) protein coverage. (**B**) Relative position of all detected HRAS bait peptides, as well as whether they were detected in unmodified, modified or with multiple delta masses. Peptides with isotopic error delta masses were considered unmodified for this representation. (**C**) Relative positions of CAL–33 bait peptides with peptides with statistical significance and fold change encoded in size and color respectively.

To demonstrate the power of our novel approach we chose to re-analyse pulldowns of exogenously expressed HRAS wild-type (HRAS^WT^) and HRAS^G12D^ constructs in 3 cell lines (PXD019469^17^), as this would represent highly expressed and stable peptidoforms characterized by a protein sequence change. The aim of the original study was to identify cancer-specific protein-protein interactions, especially those arising due to mutations in 31 frequently mutated genes, as possible therapeutic targets. By focusing on the HRAS^WT^ and HRAS^G12D^ cell lines, we found that HRAS bait proteins were highly enriched in these samples (Fig. 1B, Data 1^18^) and were detected by numerous PSMs, achieving 98% bait protein coverage (Fig. 2A, Data 2). Many of these peptides were only captured in a modified state, and thus would not have been detected using a conventional closed search (Fig. 2B, Data 3), therefore decreasing the total protein coverage. To confirm this, we searched the same data with MSFragger closed search where we identified only 2964 PSMs mapping to HRAS across all samples (Data 4), compared to 4909 PSMs identified with open search (Data 5), highlighting the power of open search to reveal more data about the peptidoforms of the bait protein. Furthermore, we showed that the bait PSMs correctly clustered samples both by cell line and HRAS mutation status, highlighting that HRAS peptidoforms captured by MSFragger varied between samples (Fig. 1C, Data 6). In total we identified 4041 proteins through 18846 peptides, of which 2887 were found in modified states. Specifically, we identified 26 peptides mapping to HRAS, which were present in multiple peptidoform states, as they were detected with various delta masses (Data 5). Among these, the LVVVGAGGVGK peptide was detected in its unmodified, carbamylated (+43), and G12D mutated (+58) forms (Fig. 1C, Data 5). The presence of the LVVVGAGGVGK + 58 G12D peptide, although not detected in the original study, validated the researchers’ experimental design and results^17^. This demonstrated that IP-MS data coupled with open search, provides additional information about the bait protein that would otherwise be missed. To systematically quantify these changes in the bait protein, we applied the SAINTexpress method^8^ for each cell line separately. We identified the peptides LVVVGAGGVGK (unmodified) and LVVVGAGGVGK (+58.00 Da G-> D substitution) as differentially present between HRAS WT and G12D cell lines (Fig. 1D-F, Data 7–9). Additionally, MSFragger localised the +58 modification on the G12 position, which is indicated in lower case in the peptide, providing further evidence and interpretability for this modification (Fig. 1D-F, Data 5). Finally, the position of differentially modified peptides could be indicative of an isoform switch, and as expected, when mapping the differential peptides between HRAS WT and HRAS G12D in CAL-33, we observed their strong localisation at the N-terminal of the protein with little differences downstream of the G12 position (Fig. 2C, Data 10). To improve confidence in differentially present peptidoforms identified in the open search, we researched the data with peptide N-terminal carbamylation and G-> D variable modification (both modifications informed by MSFragger delta mass localisation). Indeed, out of 557 LVVVGAGGVGK PSMs, 163 were of unmodified peptides, 288 of mutated peptides, with the rest being carbamylated (Data 11). These results improved confidence in the open search results and highlight the vast untapped potential of IP-MS data when open search is not used. Such positive controls highlight the power and reproducibility of our approach and its contribution in detecting differentially present sequence-based peptidoforms in IP-MS data.

### Identification of differential proteoforms in response to perturbation

**Fig. 3:**
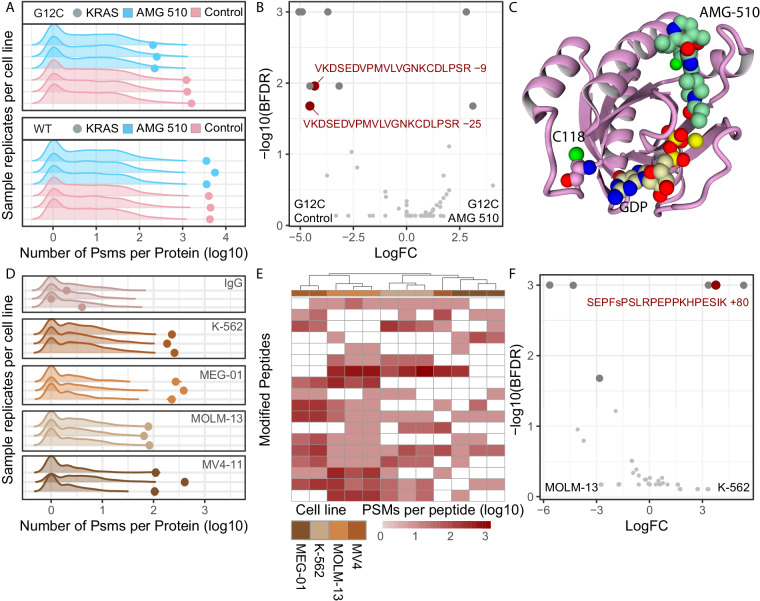
iPTMs detection of perturbation-induced and endogenous peptidoforms. (**A**) Pre-normalisation PSM counts for prey (density plots) and bait (dots) proteins for AMG-510 treated (blue) and untreated (salmon) samples for KRAS^WT^ and KRAS^G12C^ mutant cell lines, for each replicate IP-MS experiment. (**B**) Differential bait peptidoforms detected between AMG-510 and untreated KRAS^G12C^ samples according to the SAINTexpress, defined as BFDR <0.05. Only bait peptidoforms were included in the SAINTexpress analysis. (**C**) Structural model of C118 position relative to AMG-510 and GDP binding site in KRAS^G12C^ protein. (**D**) Pre-normalisation PSM counts for prey (density plots) and bait (dots) proteins for IgG control (top) and endogenously enriched BRD4 across cell lines, for each replicate IP-MS experiment. (**E**) Clustering of post-normalised PSM counts for highly abundant peptidoforms belonging to bait BRD4 protein, excluding the IgG control samples. Unsupervised clustering performed with default hclust parameters with peptidoforms not detected in specific samples assigned a zero value following log transformation. (**F**) Differential bait BRD4 peptidoforms detected between K-562 and MOM-13 samples according to the SAINTexpress, defined as BFDR <0.05. Only bait peptidoforms were included in the SAINTexpress analysis.

**Fig. 4:**
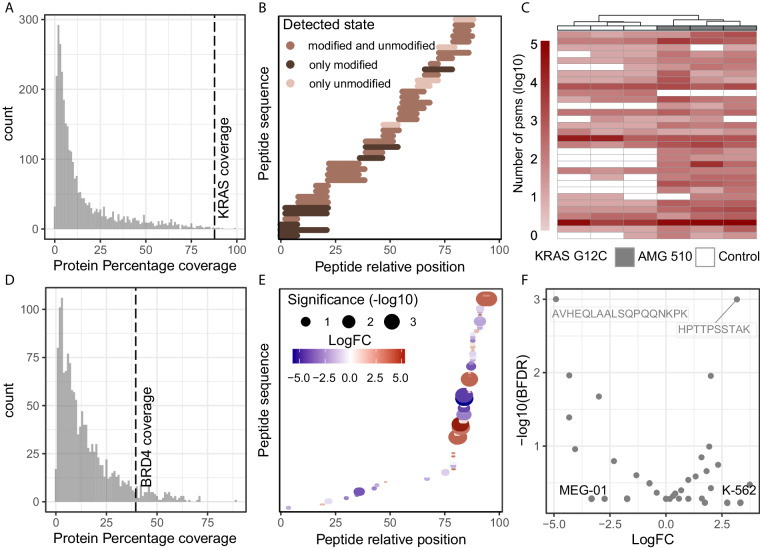
Descriptors of KRAS and BRD4 IP-MS case studies. (**A**) Percentage protein coverage for all preys and baits calculated as the percentage of amino acids detected per gene according to the longest isoform, across all KRAS IP-MS samples and all peptidoforms. Dotted line highlight the bait (KRAS) protein coverage. (**B**) Relative position of all detected KRAS bait peptides, as well as whether they were detected in unmodified, modified or in multiple delta masses. Peptides with isotopic error delta masses were considered unmodified for this representation. (**C**) Clustering of post-normalised PSM counts for highly abundant peptidoforms belonging to bait KRAS G12C protein. Unsupervised clustering performed with default hclust parameters with peptidoforms not detected in specific samples assigned a zero value following log transformation. (**D**) Percentage protein coverage for all preys and baits calculated as the percentage of amino acids detected per gene according to the longest isoform, across all BRD4 IP-MS samples and all peptidoforms. Dotted line highlight the bait (BRD4) protein coverage. (**E**) Relative positions of BRD4 bait peptides between K-562 and MOLM-13, with peptides with statistical significance and fold change encoded in size and color respectively. (**F**) Differential bait BRD4 peptidoforms detected between K-562 and MEG-01 samples according to the SAINTexpress, defined as BFDR < 0.05. Only bait peptidoforms were included in the SAINTexpress analysis.

The recent discovery and application of the KRAS^G12C^ specific inhibitors has been particularly promising. However, emerging resistance to AMG-510^13^ has already been reported^19^ and has become a new area of cancer research. Nolan *et al*.^20^ have characterised the differential interactome of KRAS^G12C^ mutated protein, aiming to understand whether the mechanism of action of the AMG-510 inhibitor, is mediated by a KRAS interactome change. To supplement this study with KRAS differential proteoform information upon the inhibitor, we investigated the differential peptidoforms of exogenously expressed KRAS^WT^ and KRAS^G12C^ upon AMG-510 treatment (PXD043536)^20^. We checked the quality of the IP-MS data by verifying that KRAS was highly enriched in the samples (Fig. 3A, Data 12) and that high protein coverage was achieved (Fig. 4A-B, Data 13-14). We identified 32 peptides mapping to KRAS, isoforms P01116-1 and P01116-2, which were present in multiple peptidoform states, suggesting a high proteoform complexity in the analysed samples (Data 15). Interestingly, the addition of AMG-510 appeared to decrease KRAS PSMs, which in the original study was attributed to the inhibitor preventing KRAS trypsinization (Fig. 3A, Data 15). WT KRAS was not strongly affected by the treatment, consistent with the specificity of AMG-510 for KRAS^G12C^ (Data 16). KRAS^G12C^ bait PSMs correctly clustered treated and control samples demonstrating that the treatment with the AMG-510 induced changes to the KRAS^G12C^ proteoform state (Fig. 4C, Data 17). Numerous modified peptides were significantly different for KRAS^G12C^ after the AMG-510 treatment (Fig. 3B, Data 18), confirming a change in KRAS^G12C^ proteoform state. Among others, the C118 position (Fig. 3C), which is known to be oxidized to allow the release of GDP and contribute to oncogenicity^15,21^, appeared to be affected by the AMG-510 treatment. In particular, C118-modified containing peptides were identified as differentially presented in the samples, being modified to cysteic acid (+47.98 Da) and to a sulfinic acid (+32.00 Da) in presence of AMG-510. The respective −9 and −25 delta mass reported by MSFragger were due to the absence of carbamidomethylation on these cysteine residues (+57.02) as a fixed modification, a chemical derivative which arises when samples are treated with iodoacetamide^22^ but that is not added when cysteines are oxidised^23^.

These data highlight the effect of drug treatments to proteoform abundance and our methodology’s ability in capturing these changes.

### Identification of endogenous proteoforms

Finally, although exogenously expressed proteins increase yield in IP-MS experiments, they can also increase false positives by changing the physiological state of the bait protein^24,25^. To validate our approach in an endogenous setting, we explored endogenous BRD4 pulldowns that we previously published in four different leukemia cell lines (PXD012715)^26^. The aim of the original study was to chatacterise novel BRD4 interactors, where we identified that methylenetetrahydrofolate dehydrogenase 1 (MTHFD1), a central enzyme of one carbon metabolism, interacts with BRD4 on chromatin, regulating nucleotide availability and cancer associated transcriptional control. In our open-search reanalysis, we observed different levels of BRD4 enrichment in the 4 different cell lines (Fig. 3D, Data 19), suggesting differential BRD4 baseline expression across them, a detail not described in our previous analysis^26^. Our analysis workflow mitigated this effect by normalizing all bait PSMs per sample to make the comparison equitable. Despite the lower protein coverage (35%, Fig. 4D, Data 20), which may be the result of endogenous pulldown rather than of an overexpressed bait, BRD4 bait PSMs correctly clustered 15 out of 16 samples, demonstrating the presence of different proteoform states between cell lines (Fig. 3E, Data 21). Among the samples we identify 1442 proteins by 6070 peptides, of which 1081 were present in modified peptidoforms. Specifically, 31 BRD4 peptides were identified, which existed in multiple peptidoform modified states, and as such would have been missed in a closed search (Data 22). Focusing on the K-562 and MOLM-13 cell lines, we identified the differential presence of a phosphorylated peptide (Fig. 3F, Data 23), which underlines the power of our approach in detecting PTMs of endogenously expressed proteins. The S1126 phosphorylation that was unbiasedly detected by MSFragger open search has been previously characterised^27^, thus increasing our confidence in its identification. Interestingly, the majority of BRD4 differential peptides seemed to map to the C-terminus of the protein with few peptides detected in the N-terminus (Fig. 4E, Data 24). Lastly, while it was possible to detect differentially present unmodified peptides (Fig. 4F, Data 25), in the absence of their modified counterpart, these peptides are harder to interpret, as it is unclear whether they represent an isoform switch, a change in cleavage site or a differential PTM occupancy of a counterpart peptidoform that was not captured.

Concluding, our novel methodology provides insight into modified peptidoforms through their enrichment in IP-MS data and provides a bridge between protein interactomes and proteoforms states.

## Discussion

Our methodology allows the capture of differentially modified peptides by analyzing antibody capture protocols. Obtaining paired interactome and proteoform changes from the same samples, opens up the possibility of integrating such information with protein-protein interaction (PPIs) interface studies to determine whether the peptidoform changes could interfere with PPIs. However, this approach is not applicable to interactome approaches that directly enrich prey proteins, such as Bio-ID^24,28^. Additionally, the open search localisation aware technology has its own limitations and often a mass shift cannot be accurately localised. As such, our analysis assigns the mass shift to the peptide rather than amino acid resolution, thus allowing the use of peptidoforms that lack amino acid localisation. The localisation and confidence of a peptidoform can be enhanced by re-searching for the significant peptidoforms as variable modifications in closed search, as demonstrated for HRAS. Given the recent developments of open search engines as a method for analysing mass spectrometry data, certain limitations have yet to be resolved by the community. Primarily, the interpretation of the mass shifts requires expert knowledge of the possible biological and chemical modifications that peptides can acquire based on the amino acid localisation of the mass shift as well as the reagents used during sample preparation. In addition, the combinatorial nature of mass shift makes larger mass shifts caused by multiple modifications of a peptide difficult to interpret. In this regard, efforts such as PTM-Shepherd^23^ are very useful for mass shift interpretation.

While our methodology complements classical interactome analysis and can be used to retrospectively analyse publicly available datasets, we hypothesize that protocols can be optimised by increasing the stringency of sample washes to shift the balance of PSMs retrieved towards the bait, enhancing the power of our methodology. Similarly, improved protocols that limit contaminants in IP-MS samples such as anti-fouling agents^29^ would improve proteoform detection. Protein and peptidoform coverage is also affected by protein length. In the HRAS example, an outstanding 98% coverage was observed, partially due to short sequence length of the bait (189 amino acids). Although not often recognised even in classical IP-MS, the degree of bait enrichment in each sample strongly affects downstream analysis, as in the demonstrated case of BRD4 expression where MV4-11 had low reproducibility in bait enrichment, thus decreasing interactor and peptidoform enrichment in some samples (Fig. 3D). In cases of drastic differences in bait enrichment between samples and conditions, spectral counting normalisation suffers, and we recommend using PSM intensity both for normalisation and the intensity-based model of SAINTexpress. Lastly, although bait PSM normalisation allows comparison between samples with different expression of the bait, such as in the case of BRD4, care should be taken when performing sample preparation to treat samples similarly to avoid batch effects and introduction of post-lysis peptidoforms. For example, N-terminal carbamylation, as seen in the HRAS peptide, might be introduced by the use of urea^30^. Samples should be treated with comparable concentrations and durations of reagents to avoid differential artifacts in the samples during preparation.

Similar to other trypsinisation-based bottom-up methodologies, our approach cannot directly determine whether the modified peptides co-occur on the same protein molecule. Finally, since bait capture is based on antibody binding to the bait, any PTMs, compounds or interactions that interfere with this binding will prevent these proteoforms from being captured, and a polyclonal capture could be considered to mitigate this.

In this study we have used the SwissProt curated protein sequences (see methods) as we were mainly interested in single point mutations and PTMs on bait proteins. In studies where the focus is alternative splicing, translational start sites, post translational cleavage or any other mechanism that could change the sequence of the bait by more than a point mutation, we propose that researchers design their own fasta file, based on possible isoforms that could exist, including any tags that have been used for the bait.

## Methods

### Data processing

Raw files were downloaded from the PRIDE proteomics repository^31^, for the HRAS (PXD019469)^17^, KRAS (PXD043536)^20^ and BRD4 (PXD012715)^26^ respectively. These data were analysed using MSFragger (FragPipe 19.2-build 11, MSFragger 3.8, philosopher 5.0.0) open search default settings^6^ with additional information about the mass shifts derived from PTM-Sheppard^23^. Human uniprot^32^ SwissProt sequences (with protein isoforms) were used for the *in silico* digestion, as downloaded on the Oct 12^th^ 2023 (fasta available on github, https://github.com/SdelciLab/iPTMs). Oxidation of methionine was included a variable modification, and cysteine carbamidomethylation was included as a fixed modification according to the original reagents and searches performed in the respective original studies.

### Differential peptide analysis

PSMs mapping to the bait protein were counted per sample per modification state with each peptide being assigned a modification mass of its reported delta mass, rounded to the closest decimal point. The MSFragger mass shift localisation and PTM-Sheppard annotation was used to explore the identity of the mass shift, with larger combinatorial modifications being much harder to characterise. To ensure enrichment differences between samples did not cause bias in the differential analysis, each sample’s total bait PSM count was normalised to the total counts of the sample with the least bait PSMs within each comparison to be made. These normalised counts were the input to SAINTexpress^8^, for differential peptide analysis. To limit false identifications of peptidoforms, we exclude from our analysis peptides with mass deltas which had a single PSM assigned to them in the whole dataset, as these would were absent in almost all conditions.

#### Sample preparation and Mass spectrometry acquisition

We provide a brief summary of publicly available datasets being re-analysed, for improved context of our analysis and results. Briefly, HRAS IP-MS samples were immunoprecipitated (Anti-Flag M2 Magnetic Beads, Sigma) from point mutant baits (via site-directed mutagenesis) and WT baits after using creation of stable cell lines (CAL-33, HET-1A and SCC-25) via lentivirus incorporation^17^. For KRAS IP-MS cells were transfected with pCEFL-FLAG-KRAS^WT^, or with pCEFL-FLAG-KRAS^G12C^. Cell lysates (with the addition of protease and phosphatase inhibitors) underwent immunoprecipitation (anti-FLAG M2 conjugated agarose beads, Sigma-Aldrich; A2220). For elution and digestion 2 M Urea was used as well as 50 mM Tris-HCl (pH7.5), trypsin and DTT (for digestion).Finally, the BRD4 IP-MS was performed on nuclear extracts using an Anti‐BRD4 (A301‐985 A, Bethyl Labs) antibody (50 µg) was coupled to 100 µl AminoLink resin (Thermo Fisher Scientific).

#### Peptidoform clustering

For a qualitative view of the detected proteoforms, we cluster the samples based on the number of PSMs per peptidoform. As with clustering in other omics datasets, we limit the features to abundantly detected peptidoforms across samples. Following Log base 2 transformation of the PSMs, we mark peptidoforms that were not detected in samples as having zero PSMs in those samples. The matrix in then clustered using pheatmap function defaults^33^, which are euclidean distance for rows and columns with ‘complete’ clustering method.

### KRAS AMG-510 structure

Figure was obtained using yasara^34^ visualization software. The model (6oim) was obtained from the Protein Data Bank^35^ and Serine 118 was mutated to Cysteine using FoldX 30874800 BuildModel command^36^. The resulting DDG of 0.81 kcal/mol demonstrates the suitability of the used model to host the wildtype Cysteine residue. The protein backbone represented as pink ribbon, Cysteine 118 is represented as atom spheres (pink carbons), as well as AMX (green carbons) and GDP (beige carbons).

## Data Availability

IP-MS raw files available at PRIDE proteomics repository^31^, for the HRAS (PXD019469)^17^, KRAS (PXD043536)^20^ and BRD4 (PXD012715)^26^ respectively. iPTMs analysis output available at https://github.com/SdelciLab/iPTMs in the v3.0 releases. All data supporting the presented figures are available in the Zenodo repository (10.5281/zenodo.11163747)^18^ and described below. Data 1: Number of PSMs per bait and prey protein in the HRAS case study. Data 2: Percentage protein coverage per protein in the HRAS case study. Data 3: HRAS protein peptides and whether they were detected in modified or unmodified state. Data 4: FragPipe closed search HRAS case study PSM output. Data 5: FragPipe open search HRAS case study PSM output. Data 6: Number of PSMs for abundant HRAS peptides across samples. Data 7–9: SAINTexpress output for CAL-33, HET-1A, SCC-25 cell lines. Data 10: Relative position of HRAS peptides and their differential presence. Data 11: FragPipe closed search HRAS output, with G12C-carbamylation variable modification. Data 12: Number of PSMs per bait and prey protein in the KRAS case study. Data 13: Percentage protein coverage per protein in the KRAS case study. Data 14: KRAS protein peptides and whether they were detected in modified or unmodified state. Data 15: FragPipe open search KRAS case study PSM output. Data 16: SAINTexpress output for KRAS WT AMG-510. Data 17: Number of PSMs for abundant KRAS peptides across samples. Data 18: SAINTexpress output for KRAS G12C AMG-510. Data 19: Number of PSMs per bait and prey protein in the BRD4 case study. Data 20: Percentage protein coverage per protein in the BRD4 case study. Data 21: Number of PSMs for abundant BRD4 peptides across samples. Data 22: FragPipe open search BRD4 case study PSM output. Data 23: SAINTexpress output for comparing K-562 and MOLM-13 cell lines. Data 24: Relative position of BRD4 peptides and their differential presence. Data 25: SAINTexpress output for comparing K-562 and MEG-01 cell lines.

## Code availability

All iPTMs code used to renalyse FragPipe and produce included figures is available at github.com/SdelciLab/iPTMs.

## References

[1] Smith LM (2013). Proteoform: a single term describing protein complexity. Nat Methods.

[2] Melani RD (2022). The Blood Proteoform Atlas: A reference map of proteoforms in human hematopoietic cells. Science (1979).

[3] Toby TK (2017). Proteoforms in Peripheral Blood Mononuclear Cells as Novel Rejection Biomarkers in Liver Transplant Recipients. Am J Transplant.

[4] Demeulemeester, N. *et al*. msqrob2PTM: differential abundance and differential usage analysis of MS-based proteomics data at the post-translational modification and peptidoform level. *Molecular & Cellular Proteomics* 100708, 10.1016/J.MCPRO.2023.100708 (2023). 10.1016/j.mcpro.2023.100708 PMC10875266 38154689

[5] Trinkle-Mulcahy L (2008). Identifying specific protein interaction partners using quantitative mass spectrometry and bead proteomes. J Cell Biol.

[6] Yu F (2020). Identification of modified peptides using localization-aware open search. Nature Communications.

[7] Kong AT, Leprevost FV, Avtonomov DM, Mellacheruvu D, Nesvizhskii AI (2017). MSFragger: ultrafast and comprehensive peptide identification in shotgun proteomics. Nat Methods.

[8] Teo G (2014). SAINTexpress: improvements and additional features in Significance Analysis of Interactome software. J Proteomics.

[9] Adams, C., Boonen, K., Laukens, K. & Bittremieux, W. Open Modification Searching of SARS-CoV-2–Human Protein Interaction Data Reveals Novel Viral Modification Sites. *Molecular and Cellular Proteomics* **21**, (2022). 10.1016/j.mcpro.2022.100425 PMC9554009 36241021

[10] Kandoth C (2013). Mutational landscape and significance across 12 major cancer types. Nature.

[11] Rhyasen, G. W. *et al*. BRD4 amplification facilitates an oncogenic gene expression program in high-grade serous ovarian cancer and confers sensitivity to BET inhibitors. *PLoS One* **13**, (2018). 10.1371/journal.pone.0200826 PMC6056044 30036377

[12] White MA (1995). Multiple Ras functions can contribute to mammalian cell transformation. Cell.

[13] Lanman BA (2020). Discovery of a Covalent Inhibitor of KRASG12C (AMG 510) for the Treatment of Solid Tumors. J Med Chem.

[14] Nakajima EC (2022). FDA Approval Summary: Sotorasib for KRAS G12C-Mutated Metastatic NSCLC. Clinical Cancer Research.

[15] Huang L, Carney J, Cardona DM, Counter CM (2014). Decreased tumorigenesis in mice with a Kras point mutation at C118. Nature Communications.

[16] Filippakopoulos P (2012). Histone recognition and large-scale structural analysis of the human bromodomain family. Cell.

[17] Swaney DL (2021). A protein network map of head and neck cancer reveals PIK3CA mutant drug sensitivity. Science.

[18] Kourtis, S. Peptidoform analysis of IP-MS data allows detection of differentially present bait proteoforms. Preprint at, 10.5281/zenodo.11163748 (2024).

[19] Salmón, M. *et al*. Kras oncogene ablation prevents resistance in advanced lung adenocarcinomas. *J Clin Invest* **133**, (2023). 10.1172/JCI164413 PMC10065067 36928090

[20] Nolan A (2023). Proteomic Mapping of the Interactome of KRAS Mutants Identifies New Features of RAS Signalling Networks and the Mechanism of Action of Sotorasib. Cancers (Basel).

[21] Kramer-Drauberg M, Ambrogio C (2021). Discoveries in the redox regulation of KRAS. Int J Biochem Cell Biol.

[22] Boja ES, Fales HM (2001). Overalkylation of a protein digest with iodoacetamide. Anal Chem.

[23] Geiszler DJ (2021). PTM-Shepherd: Analysis and Summarization of Post-Translational and Chemical Modifications From Open Search Results. Mol Cell Proteomics.

[24] Zhang K, Li Y, Huang T, Li Z (2022). Potential application of TurboID-based proximity labeling in studying the protein interaction network in plant response to abiotic stress. Front Plant Sci.

[25] Sharifi Tabar M (2022). Illuminating the dark protein-protein interactome. Cell Reports Methods.

[26] Sdelci S (2019). MTHFD1 interaction with BRD4 links folate metabolism to transcriptional regulation. Nat Genet.

[27] Hornbeck PV (2015). PhosphoSitePlus, 2014: mutations, PTMs and recalibrations. Nucleic Acids Res.

[28] Roux KJ, Kim DI, Raida M, Burke B (2012). A promiscuous biotin ligase fusion protein identifies proximal and interacting proteins in mammalian cells. Journal of Cell Biology.

[29] Van Andel E (2022). Highly Specific Protein Identification by Immunoprecipitation-Mass Spectrometry Using Antifouling Microbeads. ACS Appl Mater Interfaces.

[30] Sun S, Zhou JY, Yang W, Zhang H (2014). Inhibition of protein carbamylation in urea solution using ammonium-containing buffers. Anal Biochem.

[31] Perez-Riverol Y (2022). The PRIDE database resources in 2022: a hub for mass spectrometry-based proteomics evidences. Nucleic Acids Res.

[32] Bateman A (2021). UniProt: the universal protein knowledgebase in 2021. Nucleic Acids Res.

[33] Kolde, R. pheatmap: Pretty Heatmaps. https://CRAN.R-project.org/package=pheatmap (2019).

[34] Land H, Humble MS (2018). YASARA: A Tool to Obtain Structural Guidance in Biocatalytic Investigations. Methods Mol Biol.

[35] Berman HM (2000). The Protein Data Bank. Nucleic Acids Res.

[36] Delgado J, Radusky LG, Cianferoni D, Serrano L (2019). FoldX 5.0: working with RNA, small molecules and a new graphical interface. Bioinformatics.

